# Climate change and antibiotic resistance: A scoping review

**DOI:** 10.1111/1758-2229.70008

**Published:** 2024-09-12

**Authors:** María Fernández Salgueiro, José Antonio Cernuda Martínez, Rick Kye Gan, Pedro Arcos González

**Affiliations:** ^1^ Unit for Research in Emergency and Disaster University of Oviedo Oviedo Spain

## Abstract

This scoping review aimed to investigate the potential association between climate change and the rise of antibiotic resistance while also exploring the elements of climate change that may be involved. A scoping review was performed following the Preferred Reporting Items for Systematic Reviews and Meta‐Analyses extension for Scoping Reviews, comprehensively searching scientific literature up to 31 January 2024. Multiple databases were utilized, including MEDLINE, Web of Science and SCOPUS. Various search strategies were employed, and selection criteria were established to include articles relevant to antibiotic resistance and climate change. The review included 30 selected articles published predominantly after 2019. Findings from these studies collectively suggest that rising temperatures associated with climate change can contribute to the proliferation of antibiotic resistance, affecting diverse ecosystems. This phenomenon is observed in soil, glaciers, rivers and clinical settings. Rising temperatures are associated with a rise in the prevalence of antibiotic resistance across various environments, raising concerns for global health. However, these studies provide valuable insights but do not establish a definitive causal link between environmental temperature and antibiotic resistance. The selective pressure exerted by antibiotics and their residues in ecosystems further complicates the issue.

## Abstract 2

Rising temperatures due to climate change facilitate the proliferation of bacteria. Additionally, the presence of antibiotic residues as contaminants reduces the effectiveness of antibiotics against these bacteria.

## INTRODUCTION

The emergence of antibiotic‐resistant bacteria represents a profound and escalating public health crisis worldwide. This dire situation is further exacerbated by the extensive and often indiscriminate utilization of antibiotics across various domains, including human healthcare, animal husbandry and food production, amplifying the challenge of combating resistance to these vital drugs (Global Leaders Group on Antimicrobial Resistance, 2021).

There is a demonstrated direct relationship between antibiotic consumption and the emergence and dissemination of antibiotic‐resistant bacterial strains (Ventola, 2015). Although the most significant problems are observed in the clinical environment, environmental conditions are essential in developing and spreading resistance. In 2019, it was estimated that there were approximately 1.27 million deaths globally due to antibiotic resistance, with 929,000 of them attributable to specific resistances in *Escherichia coli*, *Staphylococcus aureus*, *Klebsiella pneumoniae*, *Streptococcus pneumoniae*, *Acinetobacter baumannii* and *Pseudomonas aeruginosa* (Antimicrobial Resistance Collaborators, 2022). By 2050, around 10 million people are projected to die each year due to bacterial infection resistance (O'Neill, 2014).

In the European Union, between 2016 and 2019, there was a significant increase in the evolution of the number of infections, attributable deaths and disability‐adjusted life years lost due to antibiotic‐resistant bacteria. In 2022, multidrug‐resistant bacteria caused 33,000 deaths annually in Europe, generating an additional healthcare expenditure of approximately 1.5 billion euros (Spanish Agency of Medicines and Health Products, 2022).

Both the World Health Organization (WHO) and other official organizations have identified global climate change as the primary factor in the spread of communicable diseases (Blair, 2018; Romanello et al., 2022). Therefore, an increase in the use of antimicrobials in humans, animals, and plants can be expected. The Global Leaders Group on Antimicrobial Resistance (GLMRA) (Global Leaders Group on Antimicrobial Resistance, 2021) has emphasized that the links between antimicrobial resistance and the climate crisis require more attention and highlights the lack of initiatives focusing on the intersection of both crises. This study aimed to explore the possible direct association between climate change and the rise in antimicrobial resistance and the elements of climate change potentially involved.

## EXPERIMENTAL PROCEDURES

### Study design

It was performed a scoping review following the Preferred Reporting Items for Systematic Reviews and Meta‐Analyses extension for Scoping Reviews (PRISMA‐ScR) (Tricco et al., 2018). It aimed to collect evidence on the potential direct relationship between climate change and antibiotic resistance. It was decided to include trials, quasi‐experimental, comparative, observational, comparative and modelling studies that reported results related to antibiotic resistance due to climate change. Case reports and commentaries were excluded.

### Search strategy and selection criteria

The bibliographic sources used to identify articles of interest for the study, published up to 31 January 2024, included MEDLINE (via the PubMed search engine), Web of Science (WOS) and SCOPUS, with no language restrictions. After defining the initial search strategy using a combination of MeSH (Medical Subject Headings) descriptors, three additional strategies were defined using free‐text language, conducted through PubMed. The strategies employed in all three information sources were as follows:

1. (Drug resistance, Microbial [Mesh]) AND (Climate change [Mesh])
2. ‘Antibiotic Resistance’ AND ‘Climate Change’
3. ([Antibiotic OR antimicrobial] AND resistance) AND environment AND (‘climate change’ OR ‘climate warming’)
4. ([Antibiotic OR antimicrobial] AND resistance) AND (‘climate change’ OR ‘climate warming’)

The search was completed by consulting reports from the National Plan against Antibiotic Resistance (PRAN) published by the Spanish Agency for Medicines and Medical Devices (AEMPS) and reports from the WHO.

Duplicate articles were identified using the Rayyan software. After excluding duplicates, two authors (MFS and JACM) independently reviewed the titles and abstracts of the results to identify potentially relevant studies for inclusion. Similarly, two authors (MFS and JACM) independently reviewed the full text of these studies. Any disagreement in selection was discussed with another author (PAG) and resolved by consensus. In addition, the reference list of all included studies was reviewed to supplement the search.

**FIGURE 1:**
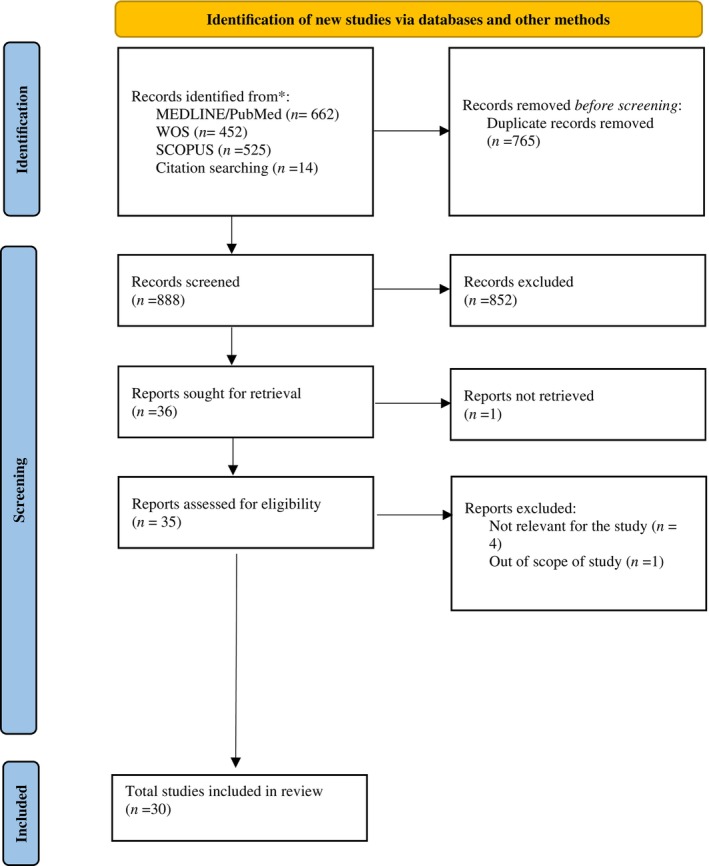
Flowchart detailing the identification, screening and selection of articles.

Articles indexed with relevant information on antibiotic resistance in relation to climate change and published up to 31 January 2024 were included. Exclusion criteria were established for articles that did not address the studied association, lacked relevant information for the study, or focused on resistance to other antimicrobials, not antibiotics, such as antifungal drugs. The flowchart detailing the identification, screening and selection of articles from the various information sources consulted is shown in Figure 1.

### Charting and extraction

For included articles, it was developed a chart on Google Sheets to extract data and confirm the relevance of full‐text articles. It was abstracted the following data: authors, year, location, title, study design, scope, objective and outcomes. Two authors (MFS and JACM) independently extracted the data of interest. Discrepancies were resolved with a third author (PAG).

### Methodological quality appraisal

Since this is a scoping review aiming to map available evidence, it was not conducted any risk of bias assessment or quality appraisal of included studies.

## RESULTS

A total of 1653 articles were identified. Of these, 765 were identified as duplicate records, and 858 were excluded from further consideration. Consequently, 30 articles were included in the review. A total of 93.3% of the articles included in the study were published from the year 2019 onwards. In descending order of geographical origin, they were from Europe (*n* = 14, including 2 from the United Kingdom), China (*n* = 6), the United States (*n* = 6), Brazil (*n* = 1), Canada (*n* = 1), Japan (*n* = 1) and one with international data. Table 1 provides the titles, first authors, publication years, study types, locations and main conclusions corresponding to the 30 selected articles included in the review. It is worth noting that despite the extensive study period with an end date of 31 January 2024, without limiting the start date, most of the articles included in this work are recent, with the highest frequency of publication in the year 2022. This increase in frequency may be due to the growing concern about the impact of climate change on antibiotic resistance.

**TABLE 1:** Summary information of the 30 articles included in the study.

Id	Reference (location)	Title	Period of study, scope, and exposure	Outcomes
1	Li, Liu, et al., 2022 (China)	Association between antibiotic resistance and increasing ambient temperature in China: an ecological study with nationwide panel data	This is an ecological and non‐population‐based study that analysed data obtained from the Antimicrobial Surveillance Network of China and other databases. The study assessed the influence of the annual average ambient temperature on the prevalence of carbapenem‐resistant *A. baumannii*, *K. pneumoniae* and *P. aeruginosa* in 28 regions of China between 2005 and 2009	The study found that the prevalence of antibiotic resistance increases with higher regional ambient temperatures and annual changes in ambient temperature have cumulative effects on antibiotic resistance
2	Mao et al., 2023 (China)	Monsoon affects the distribution of antibiotic resistome in Tibetan glaciers	This experimental and non‐population‐based study involved the analysis of 85 metagenomes from 21 glaciers and the genomes of 883 samples from Tibetan glaciers. The study aimed to identify resistance genes, mobile genetic elements and antibiotic‐resistant pathogenic bacteria. The period of study is not indicated in the article	Glaciers dominated by monsoons pose higher risks of disseminating multidrug‐resistant bacteria associated with mobile genetic elements in aquatic ecosystems when the glaciers melt
3	Yu et al., 2023 (China)	Metagenomics reveals the response of antibiotic resistance genes to elevated temperature in the Yellow River	This experimental and non‐population‐based study uses five temperatures (23, 26, 29, 32 and 35°C), employing metagenomic sequencing, measuring physicochemical properties of water, and conducting quantitative PCR of 16S rRNA. The period of study is not indicated in the article	Approximately 37% of antibiotic‐resistant genes and 42% of mobile genetic elements can be predicted by temperature. The abundance of 20 antibiotic‐resistance genes increased with temperature, and resistance to multiple drugs, tetracyclines and multi‐resistance genes showed the fastest growth
4	Bock et al., 2022 (Switzerland)	Air temperature and incidence of extended‐spectrum beta‐lactamase (ESBL)‐producing Enterobacteriaceae	This time‐series regression and population‐based study was conducted at the University Hospital in Basel, Switzerland, between 2008 and 2017. The study focused on detecting Extended‐Spectrum Beta‐Lactamase (ESBL) producing *E. coli* and *K. pneumoniae* in samples from outpatients and inpatients, as identified in the electronic database of clinical bacteriology and microbiology laboratory. The study included hourly measurements of external temperature between December 2007 and 2017. The aim was to measure the incidence risk ratio of ESBL by changes of 10°C in the external air temperature	A 10°C increase in external air temperature was associated with a relative increase in the incidence of ESBL‐producing Enterobacteriaceae of 14%–22%
5	Grenni, 2022 (Italy)	Antimicrobial resistance in rivers: a review of the genes detected and new challenges	Critical review of the scientific literature on the studied topic. It is non‐population‐based study. In this study, some recent examples of river water contaminated by antibiotics are reported	Antibiotic resistance, combined with climate change, is likely to be one of the primary crises to confront in the future
6	de Jongh et al., 2022 (Canada)	One health, one hive: a scoping review of honeybees, climate change, pollutants and antimicrobial resistance	Scoping and non‐population‐based review following PRISMA criteria. The objective of this scoping review was to examine the range, extent and nature of published literature on the relationship between AMR and honey bees in the context of climate change and environmental pollutants. The period of study is not indicated in the article	There is a need for broader research to understand the association between antimicrobial resistance, climate change, environmental pollution and the health of honeybees in the context of One Health
7	Kusi et al., 2022 (USA)	Antimicrobial resistance development pathways in surface waters and public health Implications	Narrative and non‐population‐based review. The purpose was to identify the major components of AMR pathways in surface waters and their health implications. The period of study is not indicated in the article	The threat to public health from antimicrobial resistance can grow as pathogens adapt to antibiotic residues, contaminants and the effects of climate change in aquatic systems. Unnecessary antibiotic use increases the risk of resistance, and promoting appropriate use is crucial to reduce this risk
8	Lambraki et al., 2022 (Sweden)	Governing antimicrobial resistance in a changing climate: a participatory scenario planning approach applied to Sweden in 2050	A qualitative and non‐population‐based study involved the participation of experts from multiple sectors in workshops and interviews, planning future scenarios, strategic priorities and necessary actions to address antimicrobial resistance concerning climate change. This study explored future worlds and actions that may successfully address AMR in a changing climate in a high‐income country, using Sweden as the case. The period of study is not indicated in the article	The participatory approach in scenario planning identified the need to promote global collaboration across multiple sectors to effectively address antimicrobial resistance and climate change, promote health and disease prevention, and achieve sustainable development goals to address antimicrobial resistance in a changing climate effectively. Taking immediate action to address it will help build resilience against climate change‐induced changes and ensure food, health and overall well‐being
9	Li, Sun, et al., 2022 (China)	Climate warming increases the proportions of specific antibiotic‐resistance genes in natural soil ecosystems	An experimental and non‐population‐based study was conducted to simulate climate warming by increasing the temperature by 4°C over three seasons in randomly selected groups of natural forest and plantation ecosystems in China. The period of study is not indicated in the article	Simulated climate warming increased the proportions of specific antibiotic‐resistance genes in forest soils, with a high dependence on the seasons
10	Sanchez‐Cid et al., 2022 (Poland)	Environmental and anthropogenic factors shape the snow microbiome and antibiotic resistome	An experimental and non‐population‐based metagenomic study was conducted in March 2020 using snow samples collected from areas with varying levels of anthropogenic activities and the surrounding forests in the Sudetes Mountains of Poland	Anthropogenic activity could serve as an indirect source of environmental contamination and stimulate the development of antibiotic resistance in the snow microbiome, which could subsequently spread through the atmosphere and be released as the snow melts
11	Sorn et al., 2022 (Japan)	Potential impact factors on the enhancement of antibiotic resistance in a lake environment	An experimental and non‐population‐based study was conducted using water samples obtained in December 2016 and October 2017 from Lake Kahokugata in Japan. Susceptibility testing for norfloxacin was conducted on isolated *E. coli* cultures	Organic pollution from wastewater in the lake's environment is a potential cause of increased antibiotic resistance, even at sublethal concentrations. A longer exposure time will increase the likelihood of resistance induction
12	Weldon et al., 2022 (International)	Governing global antimicrobial resistance: 6 key lessons from the Paris climate agreement	An article that compared the Paris Climate Agreement with the existing global efforts on antimicrobial resistance (AMR). The period of study is not indicated in the article	World leaders must take ambitious action to protect antimicrobials as a precious shared resource to prevent AMR and address this emerging crisis
13	Zheng et al., 2022 (China)	Metagenomics highlights the impact of climate and human activities on antibiotic resistance genes in China's estuaries	Non‐population‐based study of antibiotic resistance profiles in 16 estuaries in China during dry and wet seasons in six climatic zones and their possible relationship with environmental factors using metagenomic techniques. The period of study is not indicated in the article	A higher number and diversity of antibiotic‐resistance genes were observed during the dry season. An increase in temperature was associated with a lower number of antibiotic‐resistance genes
14	Allel, 2021 (UK)	Exploring the relationship between climate change and antimicrobial‐resistant bacteria: to what extent does this present a current and long‐term threat to population health?	A review that explores the linkage between climate change and AMR, through the role of temperature, humidity and the presence of metals in the environment. The period of study is not indicated in the article	Given that environmental changes due to climate change can favour the survival of bacteria, the health burden caused by antimicrobial resistance and climate change could worsen in the future. Governmental and population interventions are necessary for prevention
15	Burnham, 2021 (USA)	Climate change and antibiotic resistance: a deadly combination	A narrative review that discusses observations of some of the phenomena already occurring, those that are likely to occur and those that are possible as the status quo maintainers of the world continue to fail to rise to the challenge of climate change. The period of study is not indicated in the article	Climate change is a social justice issue, and its unmitigated progression will disproportionately affect the health and well‐being of people in low‐ and middle‐income countries worldwide. Antibiotic resistance and climate change are closely linked. Intense precipitation events promote bacterial mutagenesis and the expression of antibiotic‐resistance genes
16	Grilo et al., 2021 (Portugal)	Climatic alterations influence bacterial growth, biofilm production and antimicrobial resistance profiles in *Aeromonas* spp.	Non‐population‐based study of the simulation of ecosystems using freshwater samples from rivers in the Lisbon district, in pure and mixed cultures of *Aeromonas* sp. in environments with varying temperature (°C) and pH values. The period of study is not indicated in the article	Climate alterations can increase the proliferation and virulence and modulate the expression of phenotypic resistance in bacterial genera such as *Aeromonas* sp. This information is crucial for predicting and preventing future outbreaks and their harmful effects on human and animal populations
17	Güvenir et al., 2021 (Cyprus)	Do seasonal changes and climate affect the prevalence of antibiotic resistance of *Acinetobacter calcoaceticus‐baumannii* complex?	A retrospective and population‐based study of the prevalence of infections caused by antibiotic‐resistant *Actinobacter calcoaceticus‐baumannii* complex during the period from 2016 to 2019 at the Near East University Hospital in Cyprus and its potential variation with seasonality and other climatic factors	Infections caused by the *Actinobacter calcoaceticus‐baumannii* complex and carbapenem resistance of the *Actinobacter calcoaceticus‐baumannii* complex increase during the summer months
18	Harring & Krockow, 2021 (UK)	The social dilemmas of climate change and antibiotic resistance: an analytic comparison and discussion of policy implications	Narrative and non‐population‐based analysis of climate change and antibiotic resistance using game theory as a conceptual framework. The period of study is not indicated in the article	In the face of climate change and antimicrobial resistance, international binding agreements and institutions are crucial for coordinating national or local initiatives
19	Nascimento et al., 2021 (Brazil)	Amazon deforestation enriches antibiotic‐resistance genes	Experimental and non‐population‐based study of 48 soil samples from the Amazon (approximately 800 million sequences), chemical analysis, total DNA extraction, and genomic sequencing. Screening for antibiotic resistance genes. The period of study is not indicated in the article	Deforestation of the Amazon enriched the soil with antibiotic resistance genes, and anthropogenic alterations can exert selective pressure on microbial communities and expand the soil resistome
20	Pepi & Focardi, 2021 (Italy)	Antibiotic‐resistant bacteria in aquaculture and climate change: a challenge for health in the Mediterranean area	Review article on bacterial antibiotic resistance and climate change in aquaculture in the Mediterranean area. Aquaculture in the Mediterranean basin involves some critical issues, one of which is antibiotic resistance due to the use of antibiotics in animals. The emergence of antibiotic‐resistant bacterial strains and the transfer of resistance to human pathogens are noteworthy. The period of study is not indicated in the article	Two joint actions can improve public health conditions in the Mediterranean area: (1) reducing the use of antibiotics in aquaculture by improving fish care and promoting facility hygiene; (2) considering the need for actions to control climate change, with the primary intervention being the reduction of CO2 production to control temperature increases and avoid the dramatic rise of 3–4°C by 2030
21	Gudipati et al., 2020 (USA)	Can the one health approach save us from the emergence and reemergence of infectious pathogens in the era of climate change: implications for antimicrobial resistance?	Narrative review on ‘One Health’ strategies to reduce antimicrobial resistance in relation to climate change. This approach recognizes that human health is closely linked to the health of animals and environmental health. The period of study is not indicated in the article	The ‘One Health’ concept is gaining global recognition as an effective way to address health issues at the human‐animal‐environment interface. In addition to the need for communication, collaboration and coordinated activities by professionals in these sectors, the support of legislative and health systems is required to strengthen education and awareness
22	Lee et al., 2020 (USA)	Residential urban stormwater runoff: a comprehensive profile of microbiome and antibiotic resistance	Experimental and non‐population‐based study of samples collected from four wastewater outlets in the United States (Columbus, Ohio) during the spring and summer of 2017. A metagenomic approach was used to analyse microbial profiles and the resistome	Extreme precipitation contributes to high concentrations of *E. coli* in stormwater and exhibits a variable profile with mobile genetic elements. Faecal bacteria from ruminants and those associated with humans were dominant. Stormwater can contribute to the transmission of pathogens and antibiotic resistance in nearby surface waters
23	McGough et al., 2020 (Europe)	Rates of increase of antibiotic resistance and ambient temperature in Europe: a cross‐national analysis of 28 countries between 2000 and 2016	Ecological population‐based analysis of the evolution of antibiotic resistance in *E. coli*, *K. pneumoniae* and *S. aureus*, in 28 European countries. Its association with the effect of temperature and other factors was evaluated between 2000 and 2016	Evidence was found that the long‐term effect of minimum ambient temperature on antibiotic resistance increases the resistance rate in Europe. The global temperature rise can accelerate the spread of resistance, complicating mitigation efforts
24	Reverter et al., 2020 (France)	Aquaculture at the crossroads of global warming and antimicrobial resistance	Meta‐analysis to assess the influence of temperature on the mortality of aquatic animals infected with common pathogenic bacteria in fish farms; and systematic review to estimate the abundance of antibiotic resistance in aquaculture environments and record a multi‐resistance index for antibiotics for 40 countries. The period of study is not indicated in the article	Antimicrobial resistance indices in aquaculture correlate with those of bacteria in human clinical settings, temperature, and the climate vulnerability of countries. Cultivated fish exhibit higher mortalities in warm temperatures
25	Rodríguez‐Verdugo et al., 2020 (USA)	Compounding effects of climate warming and antibiotic resistance	Narrative review that synthesizes laboratory, hospital, and environmental study results on the relationship between climate change and antibiotic resistance. The period of study is not indicated in the article	Temperature changes will likely lead to thermal adaptation in bacteria, which can increase antibiotic resistance. While most bacteria are not pathogens, they serve as reservoirs of resistance genes that can be transferred to pathogens. A multidisciplinary and multi‐level approach that considers the effects of temperature is necessary
26	Kaba et al., 2020 (Germany)	Thinking outside the box: association of antimicrobial resistance with climate warming in Europe – a 30 country observational study	Cross‐sectional population‐based study in 30 European countries. The prevalence of *carbapenem‐resistant P. aeruginosa* (CRPA), *carbapenem‐resistant K. pneumoniae* (CRKB), *multidrug‐resistant E. coli* (MREC) and methicillin‐resistant *S. aureus* (MRSA) was determined over 6 years (2011–2016), along with seasonal temperature records (1991–2015). The possible association between resistance prevalence and climatic variables was analysed using bi and multivariate analysis, considering health and socioeconomic confounding factors	A novel association between antimicrobial resistance and climatic factors in Europe was identified, revealing two aspects: Climatic factors significantly explain antimicrobial resistance in different healthcare systems Climate change could increase the transmission of resistance, particularly CRPA
27	Yang et al., 2019 (China)	Bacterial community and climate change implications affected the diversity and abundance of antibiotic resistance genes in wetlands on the Qinghai‐Tibetan Plateau	Experimental non‐population‐based study in 32 heterogeneous wetlands on the Qinghai‐Tibet Plateau (covering 2463 km^2^). The study analysed environmental factors in soil samples (composition, conductivity, pH, other physicochemical characteristics and colourimetric analysis), DNA extraction, determination of genes, mobile genetic elements, etc. The period of study is not indicated in the article	Climate change and anthropogenic factors influence the profile of antibiotic‐resistant genes and their distribution across bacterial communities
28	MacFadden et al., 2018 (USA)	Antibiotic resistance increases with local temperature	An ecological non‐population‐based study of the effect of temperature and other additional factors on the distribution of antibiotic resistance in different regions of the United States between 2013 and 2015	A 10°C increase in temperature was associated with a 4.2% increase in antibiotic resistance for *E. coli*, a 2.2% increase for *K. pneumoniae*, and a 2.7% increase for *S. aureus*. These findings suggest that current projections of antibiotic resistance burden may be significantly underestimated in the face of population growth and climate change
29	Ratajczak et al., 2010 (France)	Influence of hydrological conditions on the *Escherichia coli* population structure in the water of a creek on a rural watershed	An experimental non‐population‐based study with stream water samples from the ‘Le Bébec’ basin in Upper Normandy, France, during different periods with varying hydrographic conditions (dry periods vs. rainy events). The study enumerated and determined the types of isolated *E. coli* and the presence of genes and conducted antibiotic resistance tests	The population of E. coli in the water is unstable; it depends on hydrological conditions, land use in the basin, and the origin and intensity of faecal bacteria contamination. After rainfall, an increase in faecal contamination was accompanied by a change in the distribution of phylogroups in the *E. coli* population
30	Meinen et al., 2023 (Germany)	Antimicrobial resistance in Germany and Europe – a systematic review on the increasing threat accelerated by climate change	This review shows that an increase in temperature can lead to higher rates of antibiotic resistance and an increased risk of colonization and the spread of pathogens in Germany and Europe. In addition, the number of healthcare‐associated infections increases with rising temperatures. Data suggest that antibiotic use is higher in areas with warmer average temperatures. The period of study is not indicated in the article	Universal health coverage and effective infection prevention and control measures, including reliable access to water, sanitation and hygiene and One Health antimicrobial stewardship, are needed to control the AMR pandemic worldwide. Investment in research and development of new antimicrobial drugs and vaccines is needed

The selected articles show what the main consequences of climate change are and how they affect some bacterial species that can induce antibiotic resistance, from a classical perspective and the current One Health perspective. They address both general and specific consequences for different bacterial species. Although it cannot be related to geographical patterns of resistance, due to the topic addressed in this study, it seemed appropriate to describe the geographical location of the publications. The wide geographic distribution of the origin of the articles also suggests the global nature of the concern about this serious threat to human, animal and environmental health, although without representation from countries with fewer resources, which may suffer the worst consequences of the problem. Table 1 shows the selected articles with stratification by region and population‐based or non‐population‐based studies, period of study, exposure and outcomes.

Climate change can alter ecosystems and alter the distribution of microbial communities by affecting interactions between bacteria and other organisms, potentially promoting the exchange of antibiotic resistance genes between different bacterial species. The component of the climate crisis most frequently studied in the reviewed articles was climate warming or its relation to the influence of temperature variations on antibiotic resistance in different ecosystems (Allel, 2021; Bock et al., 2022; Grenni, 2022; Grilo et al., 2021; Güvenir et al., 2021; Kaba et al., 2020; Li, Liu, et al., 2022; Li, Sun, et al., 2022; MacFadden et al., 2018; McGough et al., 2020; Reverter et al., 2020; Rodríguez‐Verdugo et al., 2020; Sorn et al., 2022; Yu et al., 2023; Zheng et al., 2022).

Regional annual changes in average ambient temperature had a cumulative effect on antibiotic resistance, with the sum of 4 years showing the greatest effect. The influence of climate warming on antibiotic resistance was also investigated in other environments such as aquatic (Grenni, 2022; Grilo et al., 2021; Sorn et al., 2022; Yu et al., 2023; Zheng et al., 2022) and soils (Li, Sun, et al., 2022; Nascimento et al., 2021; Ratajczak et al., 2010; Yang et al., 2019). For example, Yu et al. (2023), using metagenomic sequencing, explored how the gradual increase in temperature affects the profiles of antibiotic resistance genes and mobile genetic elements in water samples from the Yellow River (China). These authors found that 37% of antibiotic‐resistance genes and 42% of mobile genetic elements can be predicted based on temperature. The abundance of 20 resistance genes, including five high‐risk ones, increases with rising temperature, and multi‐resistance genes and tetracycline resistance genes increase more rapidly. They suggest that the gradual increase in temperature may reduce the diversity of antibiotic resistance genes but increase their quantity.

Extreme weather events can create conditions that favour the emergence and spread of antibiotic resistance by disrupting healthcare infrastructure, promoting population displacement and crowding, contaminating water and food supplies, disrupting antibiotic supply chains and placing stress on healthcare systems. Mitigating the impacts of climate change and building resilience in healthcare systems are important for addressing the interconnected challenges of extreme weather events and antibiotic resistance (Bock et al., 2022; Burnham, 2021; de Jongh et al., 2022; Grenni, 2022; Güvenir et al., 2021; Harring & Krockow, 2021; Kaba et al., 2020; Kusi et al., 2022; Li, Liu, et al., 2022; Li, Sun, et al., 2022; Mao et al., 2023; McGough et al., 2020; Nascimento et al., 2021; Pepi & Focardi, 2021; Reverter et al., 2020; Rodríguez‐Verdugo et al., 2020; Yu et al., 2023; Zheng et al., 2022).

## DISCUSSION

The excessive and improper use of antibiotics, including subtherapeutic dosing, has been considered the leading cause of antibiotic resistance for many years, and measures taken to reduce it were aimed at improving their use. However, it is now recognized that climate change can exert selective pressure on pathogens in various environments or ecosystems and, in relation to it, anthropogenic activities in the environment. Climate change may increase antibiotic resistance through several mechanisms: alterations in temperature, precipitation patterns and ecological systems may increase the spread of communicable diseases and their distribution and prevalence in new areas or make them more prevalent in current areas increasing demand for antibiotics, potentially leading to increased use, misuse of antibiotics and development of antibiotic resistance. Another essential component studied less frequently was the influence of extreme weather events (heavy rainfall, floods, runoff or monsoons, among others) on antibiotic resistance (Burnham, 2021; Lee et al., 2020; Mao et al., 2023).

Regarding the first component mentioned, it is worth noting four specific articles that cover different geographical areas: the study by MacFadden et al. (2018), conducted in the United States and focusing on the association of antimicrobial resistance with local temperatures; a cross‐sectional observational study in 30 European countries by Kaba et al. (2020), in which spatial and spatio‐temporal analyses have been carried out; the transnational study also in Europe (28 countries), in which an analysis of the evolution over time was carried out by McGough et al. (2020), and the study conducted in China by Li, Liu, et al. (2022). MacFadden et al. (2018) utilized an extensive public database of antibiotic resistance from hospitals, labs and units for surveillance. They also used postal codes to link it to local climate data from national databases. They studied the role of climate (temperature) and other factors in the distribution of antibiotic resistance in human pathogens (*E. coli*, *K. pneumoniae* and *S. aureus*) across the 48 contiguous states of the United States. *Resistance* was defined as the percentage of each pathogen that was not susceptible to a particular antibiotic. They found that increasing minimum temperature was associated with increased antibiotic resistance for most antibiotic classes, both oral and intravenous formulations, and across the studied pathogens. In different regions, a 10°C increase was associated with a significant increase in antibiotic resistance of 5.1%, 3.4% and 3.1% for *E. coli*, *K. pneumoniae*, and *S. aureus*, respectively. Therefore, these authors suggested that regional temperature increases and future climate change may alter and potentially accelerate the spread of antibiotic resistance. Kaba et al. (2020) conducted a similar study in Europe. They carried out an observational study to investigate the relationship between the prevalence of antimicrobial resistance in four carbapenem‐resistant bacterial species (CR); *P. aeruginosa* CR, *K. pneumoniae* CR, multidrug‐resistant *E. coli*, and methicillin‐resistant *S. aureus*, across 30 European countries. After dividing the sample of countries into two groups: (1) Northern and Western (NW), and (2) Southern and Eastern (SE), they studied the prevalence of antimicrobial resistance over 6 years. They used historical monthly mean temperature data, classifying it into (warm [May–October] and cold [January–April, November and December]), considering temperature values from 1991 to 2015. Their results align with MacFadden et al.'s results in the United States (MacFadden et al., 2018). They suggested an association between spatial temperature variability in Europe, which partially explains the variance in antimicrobial resistance in three bacterial species, including CR (*K. pneumoniae*), methicillin resistance (*S. aureus*) and MDR (*E. coli*). Based on their findings, Kaba et al. (2020) estimated that the prevalence of *P. aeruginosa* CR could double by 2039 in some countries in the NW group, which are more likely to experience more intense climate change.

Another noteworthy study regarding the association between increased antibiotic resistance and ambient temperature was conducted in European countries by McGough et al. (2020), which is in line with the study by MacFadden et al. in the United States. They used a complex database from the European Antimicrobial Resistance Surveillance Network to identify the rate variations in antimicrobial resistance in 28 European countries that could have been modulated by ambient temperature during the years 2000–2016. They assessed the impact of minimum temperature spatially and temporally and considered other factors, such as population density, and antibiotic consumption, among others. This study demonstrates that generally warmer ambient temperatures are associated with higher resistance rates for *E. coli* and *K. pneumoniae*. Similarly, Li, Liu, et al. (2022) explored the association between antibiotic resistance and regional temperature, and its variations over time in 28 regions of China from 2005 to 2019, using linear logarithmic regression models adjusted for socioeconomic, healthcare, and environmental factors. They analysed the prevalence of carbapenem‐resistant bacteria, including *A. baumannii* (ABCR), *K. pneumoniae* (KPCR) and *P. aeruginosa* (PACR), using data obtained from the China Antimicrobial Surveillance Network (CHINET). In this study, they discovered that a 1°C increase in the average ambient temperature was associated with an increase in prevalence (95% CI) of 1.14 (1.07–1.23) for KPCR and 1.06 (1.03–1.08) for PACR.

Specific studies on extreme weather phenomena included in this work are scarce (Burnham, 2021; Lee et al., 2020; Mao et al., 2023). In this regard, Burnham et al. (Burnham, 2021) stated that heavy precipitation favours bacterial mutations and the expression of antibiotic‐resistance genes. Mao et al. (2023) found that glaciers predominantly affected by monsoons are at a higher risk of spreading multi‐resistant bacteria associated with mobile genetic elements as they melt. Lee et al. (2020) studied the relationship between rainfall intensity and *E. coli* density (as an indicator of faecal contamination) in urban stormwater samples collected at four sewer system outlets in Columbus, Ohio, USA. They conducted metagenomic analysis in a subset of the samples to examine microbial profiles and the resistome. They found a significant positive relationship between *E. coli* density and rainfall intensity. The dominant bacterial phyla detected were Proteobacteria and Actinobacteria, and they identified a wide range of antibiotic resistance genes, including β‐lactam antibiotic resistance genes, which were ubiquitous. In addition to considering the relationship between antibiotic resistance and extreme weather events (heavy rainfall), it also assesses contamination from human and animal faecal bacteria. Antibiotics released into the environment can exert selective pressure on bacteria present in it, promoting the development and spread of resistance to these drugs, which can, in turn, be modulated by climate change. Antibiotic‐resistant bacteria and antibiotic‐resistance genes generated due to the use of antibiotics in humans and animals are released through excreta and reach surface waters, wastewater and soil (Spanish Agency of Medicines and Health Products, 2022).

Kusi et al. (2022) stated that healthcare facilities, agricultural environments, food and wildlife are the main vehicles for antimicrobial resistance, while antibiotic residues, heavy metals, and climate change serve as drivers of antimicrobial resistance in surface water. Other authors (Bock et al., 2022; Li, Sun, et al., 2022; Rodríguez‐Verdugo et al., 2020) also considered anthropogenic activities, these vehicles and determining factors in antibiotic resistance in relation to climate change in aquatic environments (a lake, the water of a creek on a rural watershed or wetlands). The increase in resistance to antibiotics in farmed areas can be related to the widespread and indiscriminate use of antibiotics in livestock farming and the development and spread of resistant bacteria due to exposure to antibiotics in feed, water or veterinary treatments. Antibiotic‐resistant bacteria can spread from farm animals to humans through various pathways, including direct contact with animals, consumption of contaminated meat or animal products, and environmental contamination. Workers in farm settings may be at particularly high risk of exposure to antibiotic‐resistant bacteria due to proximity to animals and their waste. The continuous use of antibiotics in animal agriculture exerts selective pressure on bacterial populations, favouring the survival and proliferation of resistant strains and the horizontal gene transfer. In addition, Nascimento et al. (2021) studied the effect of deforestation in the Amazon rainforest on antibiotic resistance, considering the changes it brings to soil characteristics. Soil changes modulated by climate change increase microbial diversity and the abundance of resistance genes.

It is essential to consider another human activity, aquaculture, whose effects can be relevant to antibiotic resistance and can be modulated by climate change. Aquaculture involves practices such as the routine use of antibiotics to prevent and treat fish to increase production. Regarding the expectations of this growing productive industrial activity, it is expected to double its production by 2030 (Pepi & Focardi, 2021). Weldon et al. (2022), Pepi and Focardi (2021) and Reverter et al. (2020) studied the impact of global warming on aquaculture. It is worth highlighting the work of Pepi and Focardi (2021) on the effects of climate change on aquaculture in the Mediterranean area in relation to antibiotic resistance. Due to its sensitivity, the Mediterranean Sea is one of the ecosystems most threatened by climate change. It represents a ‘hotspot’ in terms of climate change and antibiotic resistance in aquaculture, which can be significantly amplified in this area. An association has been found between the use of antibiotics in animals, the development of antibiotic‐resistant bacterial strains and their transfer to human pathogens. Climate change and increased temperatures lead to bacterial adaptation, resulting in the emergence of antibiotic resistance and the need to reduce antibiotic use in aquaculture. Despite the complexity of climate change processes and AMR, it will be important to closely monitor the changes in AMR over time to inform the prioritization of public health measures (Meinen et al., 2023).

### Limitations of the study

Among the study's limitations, the scarcity of studies with the necessary validity to establish causality between climate change and antibiotic resistance stands out, highlighting the need for specific randomized controlled studies. Additionally, only articles that directly addressed the relationship between climate change and AMR were selected for this scoping review. This relationship may have been addressed in articles examining healthcare‐associated infections, but these papers did not meet the stated objective of a direct relationship. Another potential limitation is the heterogeneity in methodology and types of articles included. Consequently, the impact of different exposures, such as alterations in temperature, humidity or the unjustified use of antibiotics, may be reflected in these articles.

### Conclusion

The impact of climate change, particularly the increase in temperature across different environmental settings, on antibiotic resistance has been identified as the most studied component related to climate change in the reviewed scientific literature. Additionally, rising temperatures facilitate bacterial growth and horizontal gene transfer, but this does not establish a causal association between environmental temperature and antibiotic resistance. On the other hand, antibiotics and their residues are environmental pollutants that exert selective pressure on bacteria in various ecosystems, increasing antibiotic resistance. While studies on the relationship between climate change and antibiotic resistance allow for predictions, they are not conclusive. Therefore, more precise research is needed to better understand the nature of this association.

## AUTHOR CONTRIBUTIONS

**María Fernández Salgueiro:** Conceptualization; writing – original draft; validation. **Pedro Arcos González:** Methodology; supervision; writing – review and editing. **Rick Kye Gan:** Investigation; formal analysis. **José Antonio Cernuda Martínez:** Methodology; writing – review and editing; formal analysis.

## CONFLICT OF INTEREST STATEMENT

The authors declare no conflicts of interest.

## Data Availability

Data sharing not applicable to this article.
